# PIK3CA mutation is a favorable prognostic factor in esophageal cancer: molecular profile by next-generation sequencing using surgically resected formalin-fixed, paraffin-embedded tissue

**DOI:** 10.1186/s12885-018-4733-7

**Published:** 2018-08-16

**Authors:** Tomoya Yokota, Masakuni Serizawa, Ayumu Hosokawa, Kimihide Kusafuka, Keita Mori, Toshiro Sugiyama, Yasuhiro Tsubosa, Yasuhiro Koh

**Affiliations:** 1 Division of Gastrointestinal Oncology, Shizuoka Cancer Center, 1007 Shimonagakubo Nagaizumi-cho Sunto-gun, Shizuoka, 411-8777 Japan; 2 Drug Discovery and Development Division, Shizuoka Cancer Center Research Institute, 1007 Shimonagakubo Nagaizumi-cho Sunto-gun, Shizuoka, 411-8777 Japan; 3 Department of Gastroenterology and Hematology, faculty of Medicine, University of Toyama, Toyama, Japan; 4 Pathology Division, Shizuoka Cancer Center, 1007 Shimonagakubo Nagaizumi-cho Sunto-gun, Shizuoka, 411-8777 Japan; 5 Clinical Trial Coordination Office, Shizuoka Cancer Center, 1007 Shimonagakubo Nagaizumi-cho Sunto-gun, Shizuoka, 411-8777 Japan; 6 Division of Esophageal Surgery, Shizuoka Cancer Center, 1007 Shimonagakubo Nagaizumi-cho Sunto-gun, Shizuoka, 411-8777 Japan; 7 Third Department of Internal Medicine, Wakayama Medical University, 811-1, Kimiidera, Wakayama-city, Wakayama, 641-0012 Japan

**Keywords:** Esophageal cancer, Formalin-fixed paraffin-embedded tissue, Next-generation sequencing, PIK3CA mutation, Prognostic factors

## Abstract

**Background:**

Practical and reliable genotyping procedures with a considerable number of samples are required not only for risk-adapted therapeutic strategies, but also for stratifying patients into future clinical trials for molecular-targeting drugs. Recent advances in mutation testing, including next-generation sequencing, have led to the increased use of formalin-fixed paraffin-embedded tissue. We evaluated gene alteration profiles of cancer-related genes in esophageal cancer patients and correlated them with clinicopathological features, such as smoking status and survival outcomes.

**Methods:**

Surgically resected formalin-fixed, paraffin-embedded tissue was collected from 135 consecutive patients with esophageal cancer who underwent esophagectomy. Based on the assessment of DNA quality with a quantitative PCR-based assay, uracil DNA glycosylase pretreatment was performed to ensure quality and accuracy of amplicon-based massively parallel sequencing. Amplicon-based massively parallel sequencing was performed using the Illumina TruSeq® Amplicon Cancer Panel. Gene amplification was detected by quantitative PCR-based assay. Protein expression was determined by automated quantitative fluorescent immunohistochemistry.

**Results:**

Data on genetic alterations were available for 126 patients. The median follow-up time was 1570 days. Amplicon-based massively parallel sequencing identified frequent gene alterations in TP53 (66.7%), PIK3CA (13.5%), APC (10.3%), ERBB4 (7.9%), and FBXW7 (7.9%). There was no association between clinicopathological features or prognosis with smoking status. Multivariate analyses revealed that the PIK3CA mutation and clinical T stage were independent favorable prognostic factors (hazard ratio 0.34, 95% confidence interval: 0.12–0.96, *p* = 0.042). PIK3CA mutations were significantly associated with APC alterations (*p* = 0.0007) and BRAF mutations (*p* = 0.0090).

**Conclusions:**

Our study provided profiles of cancer-related genes in Japanese patients with esophageal cancer by next-generation sequencing using surgically resected formalin-fixed, paraffin-embedded tissue, and identified the PIK3CA mutation as a favorable prognosis biomarker.

**Electronic supplementary material:**

The online version of this article (10.1186/s12885-018-4733-7) contains supplementary material, which is available to authorized users.

## Background

Esophageal cancer is one of the most aggressive types of cancer. In contrast to the predominance of adenocarcinoma in western countries, esophageal squamous cell carcinoma (ESCC) is mostly prevalent in eastern Asia, including Japan and China. Epidemiologic studies have established that cigarette smoking and alcohol consumption are strong risk factors for developing ESCC [1]. However, only small number of studies have investigated the prognostic effect of smoking and the association between the molecular characteristics and smoking status in esophageal cancer.

Despite the development of multimodality therapies, including surgical treatment with two- to three-field lymph node dissection [2], adjuvant radiotherapy, chemotherapy [3], and chemoradiotherapy [4], long-term outcome is still unfavorable, even in patients who undergo complete resection of their carcinomas [5].

To improve treatment outcome in patients with esophageal cancer, novel strategies have been developed, especially those that are molecularly targeted. Information on molecular characteristics may have novel therapeutic potential against esophageal cancer. Furthermore, their prognostic or predictive value is extremely useful not only for risk-adapted therapeutic strategies, but also for stratifying patients into future clinical trials for molecular-targeting drugs. For clinical use, practical and reliable genotyping procedures with a considerable number of samples are required. Advances in mutation testing for molecular-targeting drugs, including next-generation sequencing (NGS), have led to the increased use of formalin-fixed paraffin-embedded (FFPE) tissue. Although molecular profiling obtained from a validated comprehensive genomic assay is necessary, there is concern regarding sequencing quality or accuracy when using the DNA extracted from FFPE. We previously demonstrated that the combination strategy of quantitative PCR (qPCR)-based DNA quality assessment and uracil DNA glycosylase (UDG) pretreatment improved the accuracy of amplicon-based massively parallel sequencing (MPS) implemented with damaged DNA from FFPE [6].

The goal of this study was to evaluate the profiles of genetic alterations in esophageal cancer and to assess the effect of molecular characteristics on clinical outcome. To this end, we extensively analyzed gene expression and mutations obtained by automated quantitative fluorescent immunohistochemistry (AQUA) and MPS using archived FFPE samples from 135 esophageal cancer patients who underwent surgical resection, and correlated these results with the clinicopathological features, such as smoking status and survival outcomes.

## Methods

### Patients and tissues

Surgically resected FFPE tissue was collected from 135 consecutive patients with esophageal cancer who underwent esophagectomy at the Shizuoka Cancer Center and University of Toyama between October 2002 and November 2011. FFPE specimens were macrodissected to enrich the tumor content for DNA extraction and construction of a tissue microarray. Hematoxylin and eosin–stained slides were retrospectively collected, and presence of tumor cells was verified by experienced gastrointestinal pathologists. However, nine samples were not available for gene analysis because of insufficient tissue status or insufficient coverage for sequencing [6]. Thus, subsequent gene analysis was performed for 126 patients. This study was approved by both institutional review boards (approval number: Shizuoka Cancer Center, T23–3; Toyama University, 22–96).

### Genomic DNA extraction

Tumor samples with a diameter of 2 mm were punched out from the paraffin block and deparaffinized by 4 h incubations with xylene at room temperature. A QIAamp DNA FFPE Tissue Kit (QIAGEN, Hilden, Germany) was used to extract genomic DNA from FFPE tumors according to the manufacturer’s instructions. DNA concentration was determined using a double-stranded DNA (dsDNA) quantification kit (Quant-iT™ PicoGreen dsDNA Assay Kit, Life technologies, Carlsbad, CA), and data for each sample were previously described [6]. dsDNA was detectable in 134 of 135 samples.

### Assessment of DNA fragmentation with a qPCR-based assay and uracil DNA glycosylase (UDG) pretreatment

**Fig. 1:**
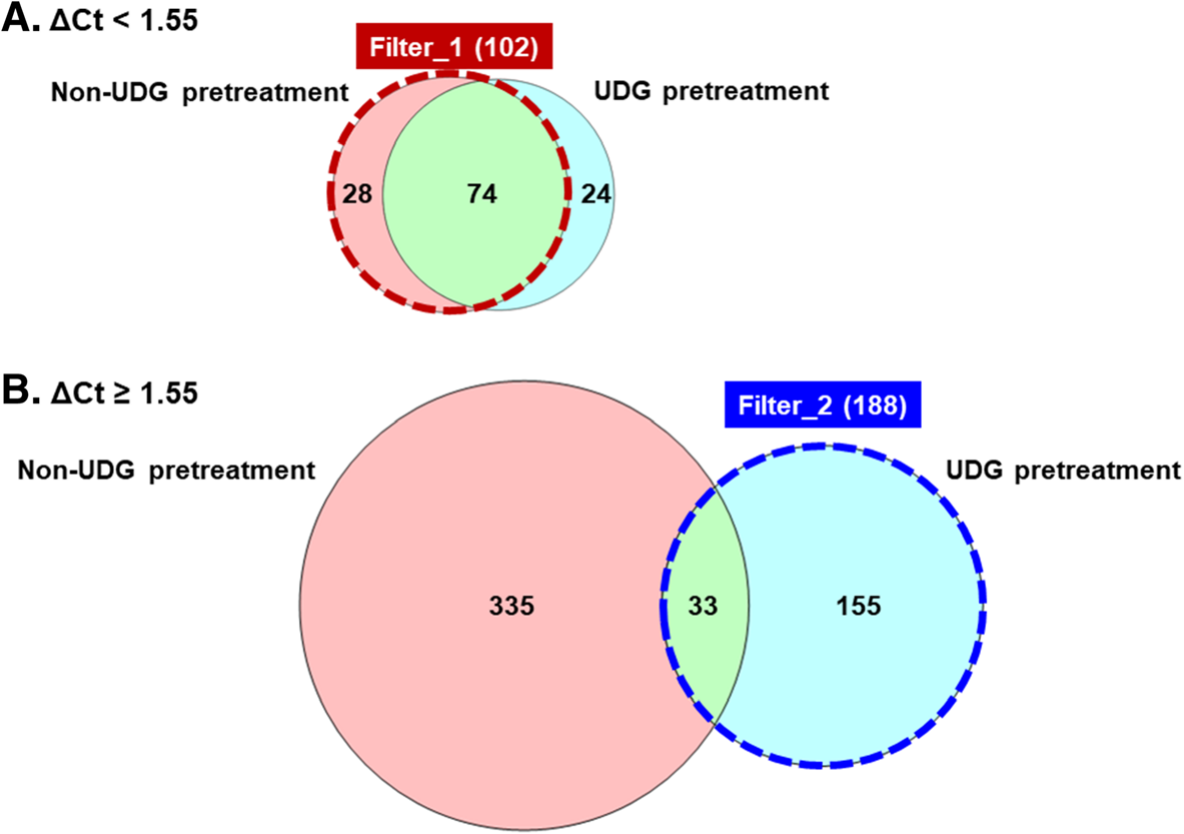
Venn diagram representing the number of nonsynonymous mutations in the samples with ΔCt < 1.55 and ΔCt ≥ 1.55. Each Venn diagram represents the number of nonsynonymous mutations reported in the COSMIC version 71 database with coverage ≥250, frequency ≥ 5%. In the samples with ΔCt < 1.55, nonsynonymous mutations with non-UDG pretreatment were selected (**a**), whereas those with UDG pretreatment were selected in the samples with ΔCt ≥ 1.55 (**b**)

A qPCR-based assessment of DNA fragmentation in 134 FFPE DNA samples was performed using the StepOnePlus™ Real-Time PCR System (Life Technologies) using 4 ng genomic DNA, SYBR® Premix Ex Taq™ II (Tli RNaseH Plus) (TAKARA BIO, Shiga, Japan), and quality check (QC) primer reagent from the Illumina FFPE QC Kit according to the manufacturer’s instructions. Cycle threshold (Ct) in amplicon-based MPS with the TruSeq Amplicon Cancer Panel (TSACP) reflects the sequencing quality in the TSACP assay. Average ΔCt values were calculated by subtracting the Ct value of the control sample from that of each sample in the three experiments. Average ΔCt values for each tumor sample were described in our previous study [6]. To ensure quality or accuracy of amplicon-based MPS, UDG pretreatment was performed using the method previously described [6, 7]. The samples with ΔCt < 1.55 were defined as acceptable sequencing quality. In 88 non-UDG pretreated samples with ΔCt < 1.55, 102 nonsynonymous mutations were detected on the basis of the human genome (hg19) CDS (coding DNA sequence) file. On the other hand, 188 nonsynonymous mutations were detected in 38 UDG pretreated samples with ΔCt of 1.55 or greater (Fig. 1).

### Amplicon-based MPS with TSACP

Amplicon-based MPS was performed on MiSeq sequencer (Illumina) using the TruSeq® Amplicon Cancer Panel (Illumina), which was designed to detect somatic mutations in 48 cancer-related genes, according to the manufacturer’s instructions. The details of data analysis for amplicon-based MPS with the TSACP assay have been described in our previous study [6]. Eight samples with less than 100× average coverage for non-UDG-pretreated or UDG-pretreated samples or both were omitted; thus, the remaining 126 samples were subjected to subsequent analysis.

### Automated quantitative fluorescent immunohistochemistry (AQUA)

A tissue microarray was constructed and protein expression levels of five representative cancer-related genes in lung and gastrointestinal tumors, including HER2, MET, EGFR, ALK, and HGF, were assessed using automated quantitative fluorescent immunohistochemistry (AQUA). The following primary antibodies were used: anti-c-erbB-2 Oncoprotein (A0485) (DAKO); anti-MET antibody (SP44); anti-EGFR antibodies (D38B1), Cell Signaling Technology; anti-ALK antibody (5A4), Abcam; and anti-HGF antibody (7-2), Abcam. Mouse IgG2a (Abcam), rabbit polyclonal IgG (Abcam), and normal goat IgG (Santa Cruz Biotechnology) were used as corresponding control antibodies. The AQUA method of quantitative immunofluorescence used to quantitatively measure the biomarkers has been previously described [8]. In brief, monochromatic, high-resolution images were obtained of each histospot after immunofluorescent staining as described herein. Images were captured by the PM-2000 microscope (HistoRx). We distinguished areas of tumor from stromal elements by creating a mask from the cytokeratin signal. A tumor nucleus–specific compartment was created by using the 4′,6-diamidino-2-phenylindole (DAPI) signal to identify nuclei, and the cytokeratin signal to define the cytoplasm and membrane. The target signal (AQUA score) was expressed as pixel intensity divided by the target area (tumor nuclei compartment). AQUA scores for triplicate tissue cores were averaged to obtain a mean AQUA score for each tumor. The AQUA scoring was a blind clinical procedure.

### Statistical analysis

The relationships between clinicopathologic variables and smoking status or PIK3CA status were assessed using Fisher’s exact test. The Wilcoxon rank sum test was used for analysis of continuous variables. Overall survival (OS) was calculated from the date of surgery until death from any cause, or censored at last follow-up visit. To investigate the prognostic factors, we performed multivariate analysis with the Cox proportional hazard model. The cutoff of protein expression was set to the median AQUA score in multivariate analysis. All *p*-values were two-tailed and *P* < 0.05 was considered statistically significant. We conducted all the analyses using R version 3.2.3 (The R Foundation for Statistical Computing, Vienna, Austria).

## Results

### Association of smoking status with clinicopathological features

Cumulative smoking dose was evaluated as pack-years (PY), the product of the number of packs consumed per day and years of smoking. In this study, subjects were categorized into four groups based upon PY: smoking status 0: nonsmoker, 1: 0 < PY < 20, 2: 20 < PY < 40, 3: 40 < PY. We then correlated the smoking habit with clinicopathological features of esophageal cancer, including age, gender, primary tumor location, histological findings, TNM stage (UICC 6th), and adjuvant therapy. Females were more frequent in the smoking status 0 group than in other groups. Furthermore, 7.1% (1 out of 14) of the primary tumors with the smoking status 0 group were located on the cervical esophagus whereas the frequencies of cervical esophageal cancer were 0%, 3.3% (1 out of 30), and 0%, for the smoking status 1, 2, and 3 group, respectively. However, there was no association between smoking status and age, histology, TNM stage, or adjuvant therapy (Table 1).

**Table 1:** Patient characteristics according to smoking history (*n* = 126)

Smoking status	0 (*n* = 14)	1 (*n* = 22)	2 (*n* = 30)	3 (*n* = 60)	*p*-value
Median age (range)	63 (44–73)	64 (46–75)	63 (42–76)	64 (46–79)	** 0.3949
Gender					
Male	10	20	25	57	*0.04537
Female	4	2	5	3	
Location					
Ce	1	0	1	0	*0.02068
Ut	4	3	1	6	
Mt	4	6	18	21	
Lt	5	13	7	28	
Ae	0	0	3	5	
Histology					
SCC	14	21	28	53	*0.642
Others	0	1	2	7	
TNM (UICC6th)					
T1	1	2	0	2	*0.163
T2	2	2	0	2	
T3	11	18	30	54	
T4	0	0	0	2	
N0	4	5	11	10	*0.1923
N1	10	17	19	50	
M0	11	18	24	51	*0.7328
M1a	2	2	1	4	
M1b	1	2	5	5	
Adjuvant					
NAC	6	11	17	31	*0.8629
No NAC	8	11	13	29	
HER2					
> median	4	12	17	31	*0.357
< median	10	10	13	29	
MET					
> median	5	13	16	33	*0.5689
< median	9	9	14	27	
EGFR					
> median	6	10	14	32	*0.8578
< median	8	12	16	28	
ALK					
> median	6	14	13	29	*0.4895
< median	8	8	17	31	
HGF					
> median	4	9	14	34	*0.2289
< median	10	13	16	26	
TP53					
Wild type	7	8	7	20	*0.372
Mutation	7	14	23	40	
APC					
Wild type	14	20	26	53	*0.6702
Mutation	0	2	4	7	
PIK3CA					
Wild type	14	16	28	54	*0.06858
Mutation	0	6	2	6	
FBXW7					
Wild type	14	19	27	56	*0.516
Mutation	0	3	3	4	
BRAF					
Wild type	14	20	28	55	*0.8769
Mutation	0	2	2	5	

Cumulative smoking dose: pack-years (PY) = Packs/day × years of smoking

Smoking status 0: non-smoker, Smoking status 1: 0 < PY < 20, Smoking status 2: 20 < PY < 40, Smoking status 3: 40 < PY

*Abbreviation: Ce* cervical esophageal cancer, *Ut* upper thoracic esophageal cancer, *Mt* middle thoracic esophageal cancer, *Lt* lower thoracic esophageal cancer, *Ae* abdominal esophageal cancer, *SCC* squamous cell carcinoma, *NAC* neoadjuvant chemotherapy

* Fisher’s exact test, ** Wilcoxon test

### Mutational analysis by TSACP

**Fig. 2:**
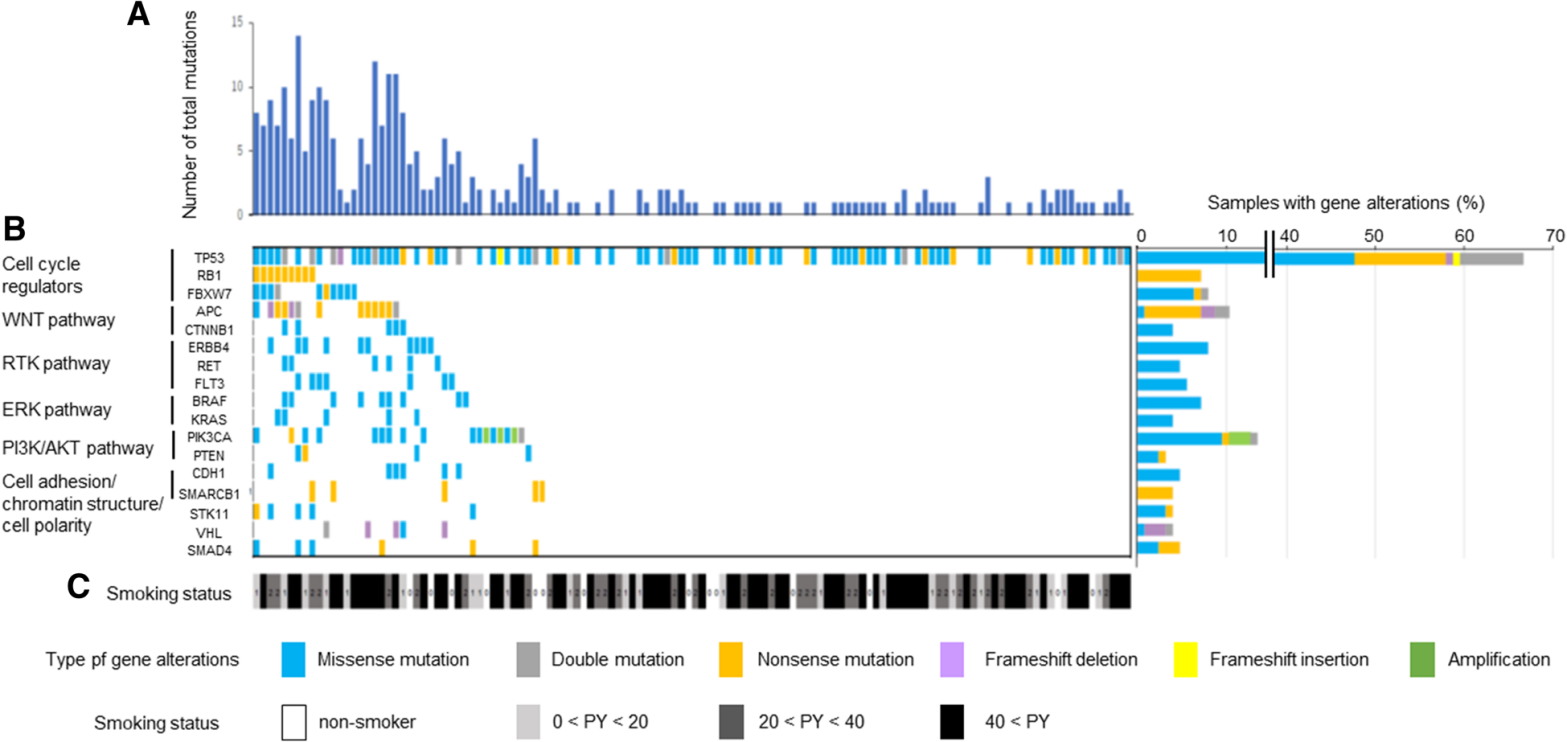
Genome-wide mutational landscape of ESCC identified by whole-exome sequencing (*n* = 126). **a** Number of total nonsynonymous mutations in individuals is illustrated in the bar graph. **b** Left, mutations in a selection of frequently mutated genes, arranged vertically by functional group and colored by the type of gene alteration. Samples are displayed as columns. Right, the percentage of individuals with somatic alterations that targeted each gene. **c** Smoking status is represented by cumulative exposure doses measured by pack years

Mutation analysis was not successful in eight cases because of poor sample condition. Thus, data on genetic alterations were available for 126 patients. Somatic mutational analysis by TSACP identified 290 gene alterations, including single nucleotide variants, deletions, and insertions. The most frequently altered gene was TP53 (mutated in 66.7% of our cohort), followed by PIK3CA (13.5%), APC (10.3%), ERBB4 (7.9%), FBXW7 (7.9%), BRAF (7.1%), RB1 (7.1%), FLT3 (5.6%), RET (4.8%), CDH1 (4.8%), SMAD4 (4.8%), VHL (4.0%), CTNNB1 (4.0%), KRAS (4.0%), SMARCB1 (4.0%), STK11 (4.0%), and PTEN (3.2%) (Fig. 2). Most of these genes exhibited missense mutations, followed by nonsense mutations and frameshift mutations, suggesting their tumor suppressor roles. Of all gene alterations in PIK3CA (*n* = 17), gene amplification was detected in three patients (2.4%), and PIK3CA mutations occurred in 14 patients (11.1%). Of all PIK3CA alterations, 58.8% were identified in three known hotspots, E542K/E542V (17.6%) and E545K (23.5%) in exon 9, and H1047R/H1047L (23.5%) in exon 20. Both E542V missense mutations and the frameshift deletion in H554 were detected in one case. There were 17 APC mutations in 13 patients (10.3%), including the nonsense mutation alone (*n* = 8), missense mutation alone (*n* = 1), both frameshift deletion and missense mutation (*n* = 1), and both frameshift deletion and nonsense mutation (*n* = 1) (Fig. 2b). Gene alterations in RB and SMARCB1 were all nonsense mutations. All raw data on somatic mutational analysis by TSACP are shown in Additional file 1.

### Effects of smoking status on gene expression and mutation profile

**Fig. 3:**
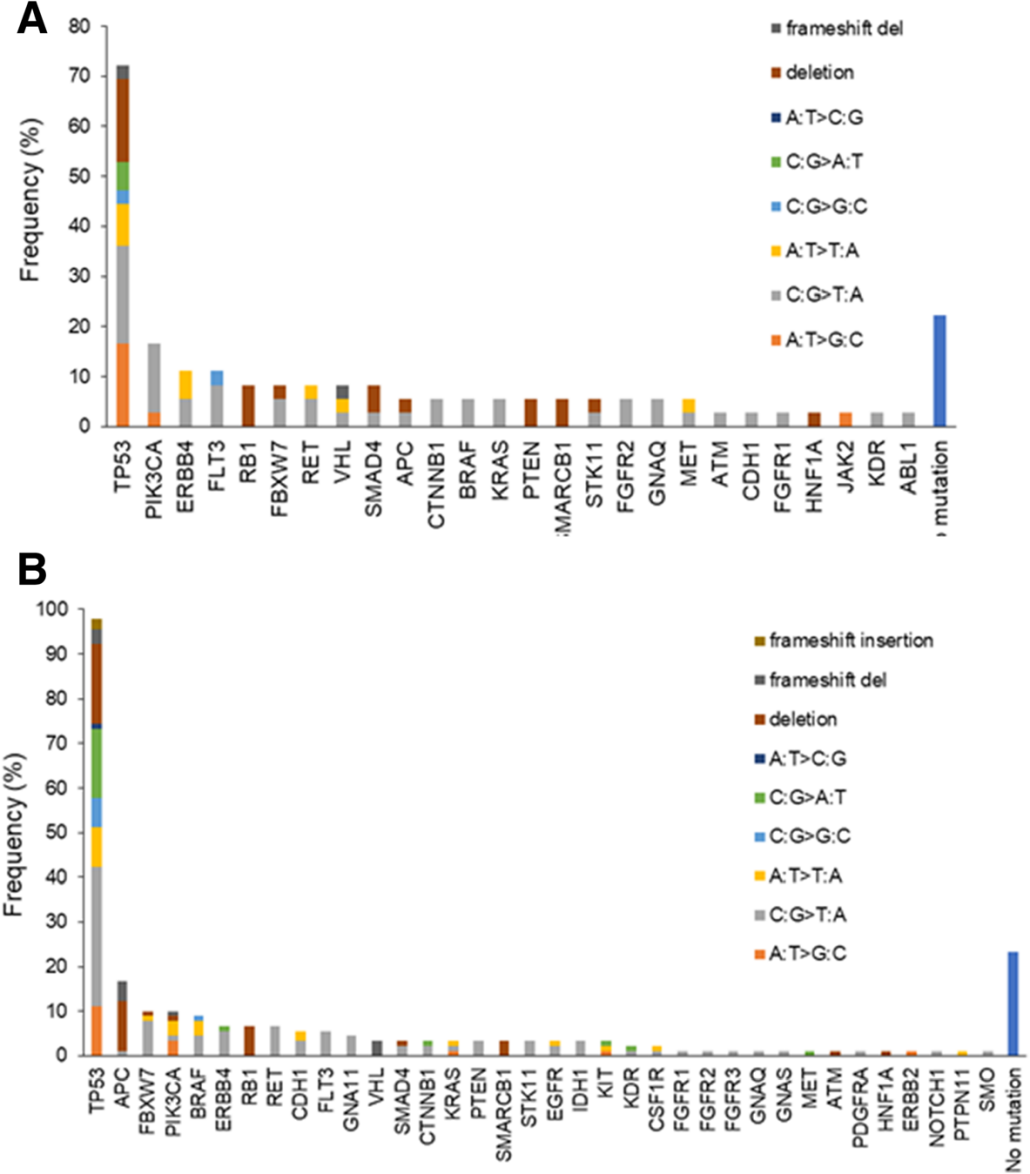
Frequency of somatic mutations with base substitution in non/light smoker (*n* = 36) and smokers (*n* = 90). Frequencies of somatic mutations are shown for indicated genes in (**a**) non/light smokers (smoking status 0 + 1) and (**b**) smokers (smoking status 2 + 3). Deletions, insertions, and six types of point mutations are differentially shown by colors

Median AQUA scores were used as the cut-off point for each protein expression. The association of smoking status with AQUA scores of target gene expression was analyzed by Fisher’s exact test. The results revealed no associations between AQUA scores for HER2, MET, EGFR, ALK, and HGF, and smoking status for the entire cohort. The association between smoking status and somatic mutations with base substitutions were further investigated. Although no PIK3CA mutation was observed among non-smokers, no significant correlation between smoking status and gene alteration was observed (Table 1, Fig. 2c). Regardless of smoking status, the most frequent mutation was TP53 (72% in non/light smoker, 98% in smokers). In non/light smoker (smoking status 0 + 1, *n* = 36), PIK3CA (17%), ERBB4 (11%), FLT3 (11%), RB1 (8%), and FBXW7 (4%) were most frequent, whereas in smokers (smoking status 2 + 3, *n* = 90), they were APC (17%), FBXW7 (10%), PIK3CA (10%), and BRAF (9%). No significant differences were found in either composition of mutations or the pattern of base substitutions between smokers and non-smokers (Fig. 3a and b).

### Prognostic factors in multivariate analyses

**Fig. 4:**
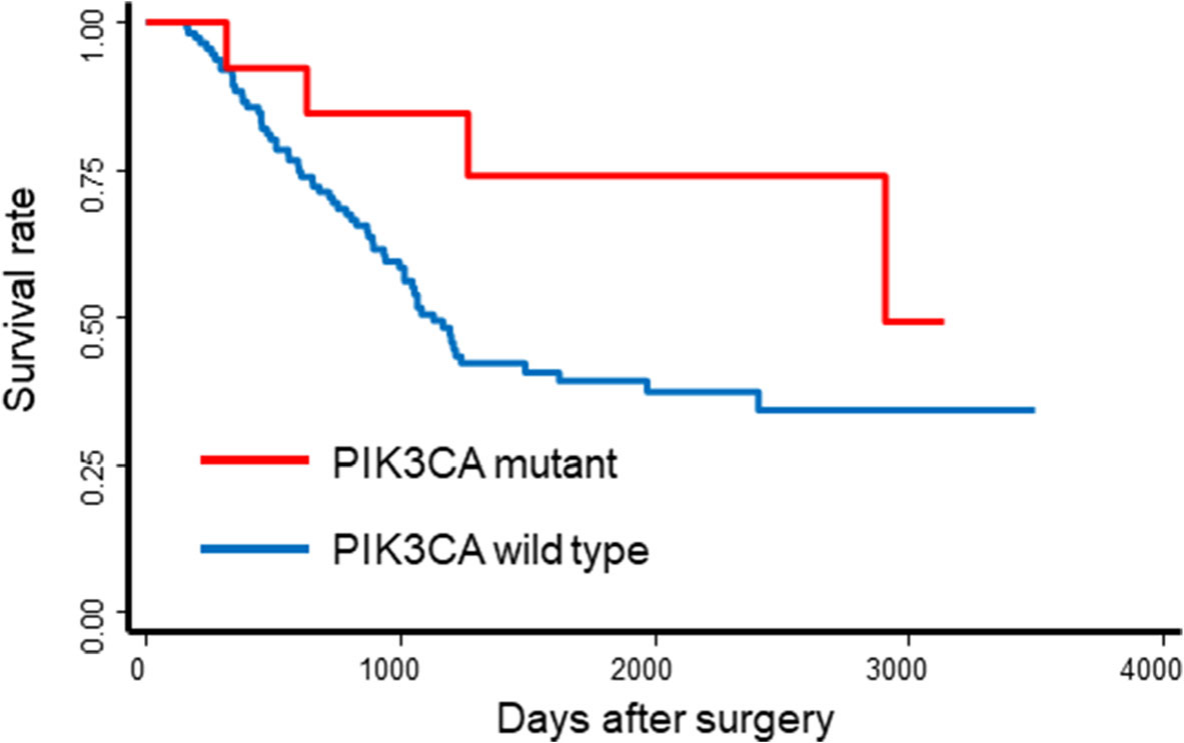
Kaplan-Meier plot showing overall survival in esophageal cancer patients according to PIK3CA mutational status (*n* = 126)

The median follow-up time was 1570 days. Univariate analysis of OS was performed using clinicopathological variables, aforementioned protein expression, and frequently mutated gene status in TSACP. A factor that was significantly statistically associated with poor OS in this analysis was clinical T stage (*p* = 0.044). Male gender and the p53 mutation were marginally statistically associated with poor OS in all patients (hazard ratio (HR) 2.36; *p* = 0.096, HR 1.72; *p* = 0.059, respectively). However, patients with PIK3CA mutations had better OS (median OS 2902 days, 95% CI 1264 days–not reached) than patients with wild-type PIK3CA (median OS 1129 days, 95% CI 938–1622 days; *p* = 0.077 (Table 2, Fig. 4). To adjust for significant prognostic factors, a Cox proportional hazard model that included all factors mentioned above was used. Clinical T stage was confirmed as an independent negative prognostic factor, whereas the PIK3CA mutation was an independent favorable prognostic factor. Multivariate analysis, including variables whose *p*-value was less than 0.1 in univariate analysis also confirmed that clinical T stage and the PIK3CA mutation were independent prognostic factors. Specifically, the HR for patients with cT3 was 4.30 (95% CI: 1.04–17.70) compared to patients with cT1 and 2 (*p* = 0.044). Furthermore, the HR for patients with the PIK3CA mutation was 0.34 (95% CI: 0.12–0.96) compared with patients with the wild-type PIK3CA (*p* = 0.042) (Table 2). However, the prognostic value of PIK3CA amplification was not statistically significant (HR 2.66; 95% CI 0.64–11.05; *p* = 0.177), and this probably occurred because of the limited number of patients with the PIK3CA amplification (*n* = 3).

**Table 2:** Factors associated with overall survival in univariate and multivariate analyses

		Univariate analysis		Multivariate analysis (including all variables)		Multivariate analysis	
Factors	Category	HR (95% CI)	*p*-value	HR (95% CI)	*p*-value	HR (95% CI)	*p*-value
Age	≥65 (vs. < 65)	1.11 (0.68–1.79)	0.68	1.25 (0.74–2.11)	0.40		
Male	Male (vs. Female)	2.36 (0.86–6.49)	0.096	2.48 (0.86–7.20)	0.094	2.30 (0.83–6.36)	0.11
Smoking status	2–3 (vs 0–1)	1.22 (0.71–2.13)	0.47	0.74 (0.40–1.36)	0.33		
cT	T3 (vs. T1, T2)	4.25 (1.04–17.41)	0.044	5.23 (1.19–23.00)	0.029	4.30 (1.04–17.70)	0.044
cN	N1 (vs. N0)	1.52 (0.84–2.75)	0.16	1.12 (0.57–2.19)	0.75		
NAC	Without (vs. with)	1.01 (0.62–1.65)	0.97	0.88 (0.52–1.50)	0.65		
HER2	>median (vs. <median)	0.93 (0.57–1.50)	0.76	0.92 (0.54–1.55)	0.75		
MET	>median (vs. <median)	0.75 (0.46–1.21)	0.24	0.74 (0.44–1.27)	0.28		
EGFR	>median (vs. <median)	0.99 (0.61–1.60)	0.97	0.95 (0.56–1.62)	0.86		
ALK	>median (vs. <median)	1.17 (0.72–1.89)	0.52	1.14 (0.68–1.92)	0.61		
HGF	>median (vs. <median)	1.29 (0.80–2.09)	0.29	1.34 (0.77–2.33)	0.29		
p53	Mutation (vs. wild type)	1.72 (0.98–3.03)	0.059	1.51 (0.82–2.81)	0.19	1.55 (0.88–2.73)	0.13
PIK3CA	Mutation (vs. wild type)	0.40 (0.14–1.10)	0.077	0.28 (0.09–0.90)	0.033	0.34 (0.12–0.96)	0.042
APC	Mutation (vs. wild type)	0.87 (0.40–1.90)	0.72	0.81 (0.23–2.86)	0.74		
ERBB4	Mutation (vs. wild type)	0.63 (0.23–1.73)	0.37	0.91 (0.27–3.10)	0.88		
FBXW7	Mutation (vs. wild type)	0.56 (0.20–1.54)	0.26	0.43 (0.13–1.44)	0.17		
BRAF	Mutation (vs. wild type)	0.99 (0.40–2.47)	0.99	1.83 (0.56–6.04)	0.32		
RB1	Mutation (vs. wild type)	1.03 (0.41–2.57)	0.95	1.79 (0.52–6.11)	0.35		

*Abbreviations: HR* hazard ratio, *CI* confidence interval, *cT* clinical T, *cN* clinical N

### Associations between PIK3CA mutations and clinicopathological factors

We then evaluated the clinicopathological and molecular characteristics of PIK3CA mutations in esophageal cancer. APC gene alterations occurred more frequently in the PIK3CA mutation than in the wild-type (*p* = 0.0007). BRAF mutations also statistically significantly occurred with the PIK3CA mutations (*p* = 0.0090). However, there was no significant relationship between the PIK3CA mutations and other clinicopathological characteristics (Table 3).

**Table 3:** PIK3CA status and associated clinicopathological factors (*n* = 126)

Variable	PIK3CA mutant		PIK3CA wild type		
	N	%	N	%	*p*-value
Age					
< 65	7	50.0	63	56.3	0.7776
≥ 65	7	50.0	49	43.8	
Gender					
Male	13	92.9	99	88.4	1.0000
Female	1	7.1	13	11.6	
Smoking status					
0	0	0.0	14	12.5	
1	6	42.9	16	14.3	0.06858
2	2	14.3	28	25.0	
3	6	42.9	54	48.2	
Location					
Ce	0	0.0	2	1.8	0.9269
Ut	1	7.1	13	11.6	
Mt	6	42.9	43	38.4	
Lt	7	50.0	46	41.1	
Ae	0	0.0	8	7.1	
Histology					
SCC	14	100.0	102	91.1	0.6007
Others	0	0.0	10	8.9	
TNM (UICC6th)					
T1	0	0.0	5	4.5	0.2775
T2	1	7.1	5	4.5	
T3	12	85.7	101	90.2	
T4	1	7.1	1	0.9	
N0	4	28.6	26	23.2	0.7402
N1	10	71.4	86	76.8	
M0	11	78.6	93	83.0	0.4465
M1a	2	14.3	7	6.3	
M1b	1	7.1	12	10.7	
Adjuvant					
NAC	7	50.0	58	51.8	1.0000
No NAC	7	50.0	54	48.2	
HER2					
> median	9	64.3	55	49.1	0.3969
< median	5	35.7	57	50.9	
MET					
> median	6	42.9	61	54.5	0.5715
< median	8	57.1	51	45.5	
EGFR					
> median	5	35.7	57	50.9	0.3969
< median	9	64.3	55	49.1	
ALK					
> median	7	50.0	55	49.1	1.0000
< median	7	50.0	57	50.9	
HGF					
> median	6	42.9	55	49.1	0.7796
< median	8	57.1	57	50.9	
TP53					
Wild type	4	28.6	38	33.9	0.7728
Mutation	10	71.4	74	66.1	
APC					
Wild type	8	57.1	105	93.8	0.0007
Mutation	6	42.9	7	6.3	
ERBB4					
Wild type	12	85.7	104	92.9	0.3070
Mutation	2	14.3	8	7.1	
FBXW7					
Wild type	12	85.7	104	92.9	0.3070
Mutation	2	14.3	8	7.1	
BRAF					
Wild type	10	71.4	107	95.5	0.0090
Mutation	4	28.6	5	4.5	
RB1					
Wild type	11	78.6	106	94.6	0.0618
Mutation	3	21.4	6	5.4	

## Discussion

In this study, we determined the genetic profiles of 126 Japanese esophageal cancer patients by NGS and AQUA using DNA from FFPE samples. Our cohort was non-biased consecutive cases, which consisted mostly of ESCC, but also of those with non-ESCC histology. Amplicon-based MPS identified mutations in TP53, PIK3CA, APC, ERBB4, and FBXW7 as the most frequent gene alterations. We further examined the prognostic effect of these gene mutations, and found that the PIK3CA mutation, as well as the clinical T stage were independent prognostic factors. Importantly, patients with the PIK3CA mutations had significantly better survival than those with the wild-type PIK3CA. To the best of our knowledge, the present report is the first to investigate the prognostic significance of PIK3CA mutations based on NGS data in esophageal cancer.

One of the goals in this study was to characterize the smoking status in esophageal cancer. However, there was no association between clinicopathological features or prognosis and smoking status. Furthermore, our molecular analysis by NGS suggested there were no significant differences in the mutation spectrum and the pattern of base substitutions between smokers and non-smokers, unlike that of non-small-cell lung cancer patients who underwent surgery [9]. These results are consistent with previous exome sequencing in ESCC from China [10], and support the hypothesis that smoking might contribute to tumorigenesis of esophageal cancer through distinct mechanisms similar to those in other smoking-related cancers.

PIK3CA, which encodes the p110a catalytic subunit of the phosphoinositide 3-kinase (PI3K) [11, 12], is an oncogene in various cancers, and its mutation or amplification and subsequent activation of the PI3K/AKT signaling pathway regulates cell proliferation, growth, survival, apoptosis, and glucose metabolism [13]. The frequency of PIK3CA mutations has been reported to range from 1.5 to 22.9% in ESCC [10, 14–23], as well as in Barrett’s esophagus [24]. In our study, 13.5% of cases were identified as having a PIK3CA mutation or amplification, all of which presented squamous cell carcinoma histology. Therefore, PIK3CA serves as a potential therapeutic target in ESCC. Hotspot mutations of PIK3CA in exon 9 and exon 20 are known to be oncogenic in various tumor types, including esophageal, colorectal, brain, and gastric cancers [25]. PIK3CA mutations were not significantly associated with any clinicopathological characteristics, except for the APC and BRAF genotype as discussed below, which was consistent with the results of other studies in Korea, China, and Japan [17, 19, 26].

The prognostic relevance of PIK3CA mutations has been investigated in various solid tumors, and PIK3CA mutations are generally associated with an unfavorable prognosis in patients with colorectal [27–30] or lung cancer [31]. On the other hand, studies on breast and ovarian cancer demonstrated that the patients with the PIK3CA mutation showed a trend towards a favorable prognosis [32–34]. These reports suggest that PIK3CA mutations might behave differently according to tumor type. Our multivariate analysis revealed that PIK3CA gene mutations were associated with a favorable prognosis among Japanese patients with curatively resected esophageal cancer, mainly ESCC, suggesting that the PIK3CA gene mutational status may be a prognostic biomarker for Japanese esophageal cancer patients. This finding supports other studies in Chinese and Japanese ESCC patients [16, 19, 22]. We further separately analyzed the survival in patients with PIK3CA mutations in coding exon 9 and 20. Median OS in patients with exon 9 mutation was not reached, and that in patients with exon 20 mutation was 2902 days (95% CI 693 days–not reached). That is, both patients with exon 9 and 20 mutation had better OS than patients with wild-type PIK3CA. These findings suggest that both exon 9 and 20 mutation might be favorable prognostic factors. However, due to limited sample size for each type of PIK3CA mutation (6 patients in exon 9, 7 patients in exon 20, and 1 in exon 7), it is hard to differentially conclude the significance of each mutation as a prognostic marker. Taken together, the prognostic effect of the PIK3CA mutation in ESCC has been controversial, despite a number of investigations dating from the 2010s in Asia (Table 4).

**Table 4:** Prognostic significance of PIK3CA alterations in esophageal cancer

Author	Sample size (N)	Histology	Type of PIK3CA alterations	Frequency (%)	Prognostic effect	HR (95% CI)	Material	Method
Maeng et al. (2012) [17]	80	ESCC	Exon 9 and 20 mutations	11.5	N.S.	NA	Primary& Metastatic sites FFPE	Mass-spectrometry based assay
Shigaki et al. (2013) [19]	219	ESCC	Exon 9 and 20 mutations	21	Favorable OS	0.35 (0.10–0.90)	Surgically resected FFPE	Pyrosequencing
Hou et al. (2014) [16]	96	ESCC	Exon 9 and 20 mutations	12.5	Trend towards favorable OS	NA	Surgically resected FFPE	Mutant enriched PCR method
Wang et al. (2014) [21]	406	ESCC	Exon 9 mutations	7.4	N.S.	1.256 (0.748–2.108)	Surgically resected FFPE	Direct sequencing
Kim et al. (2016) [23]	534	ESCC	Amplification	10.5	Trend towards unfavorable OS	1.21 (0.83–1.77)	Surgically resected FFPE	Fluorescent in situ hybridization
	388		Exon 9 and 20 mutations	1.5	N.S.	NA		Direct sequencing
Liu et al. (2017) [22]	210	ESCC	Exon 9 mutations	22.9	Favorable OS	NA	Surgically resected FFPE	Pyrosequencing
Current study	126	Mostly ESCC	Exon 9 and 20 mutations	11.1	Favorable OS	0.34 (0.12–0.96)	Surgically resected FFPE	MPS with TSACP
			Amplification	2.4	N.S.	2.66 (0.64–11.05)		

*Abbreviations: OS* overall survival, *HR* hazard ratio, *CI* confidence interval, *NA* not available, *FFPE* formalin-fixed paraffin-embedded, *N.S*. not significant, *ESCC* esophageal squamous cell carcinoma, *MPS* massively parallel sequencing, *TSACP* TruSeq® Amplicon Cancer Panel

The possible reasons for the different results might be different patient cohorts, sample sizes, methods used to assess PIK3CA mutations, or ethnicity. First, the patient cohort in Maeng et al. had metastatic ESCC, which differed from those studies using surgically resected primary sites [17]. Next, we used amplicon-based MPS, which is a NGS technology and increasingly being used for mutational analysis of tumors for both clinical and research applications. NGS facilitates multi-gene mutational profiling with only nanograms of DNA and has better sensitivity than traditional sequencing platforms, such as direct sequencing [35, 36]. Indeed, the limited sensitivity of direct sequencing may result in an apparent the low frequency of PIK3CA mutations [21, 23]. Although allele-specific mutation testing, including pyrosequencing and mass-spectrometry based assays, was shown to be more sensitive than regular direct sequencing, its potential disadvantage is the ability to assess only limited hotspot regions in given genes [37]. Therefore, different sequencing methodologies may have an effect on the frequency of PIK3CA mutations, leading to different prognostic values. Furthermore, although FFPE samples are the most practically available material when performing mutation testing, one of the pitfalls of using FFPE samples is DNA fragmentation [38] and artificial C: G > T: A single nucleotide variants because of deamination of cytosine to uracil. Therefore, DNA quality assessment is essential in mutation testing, especially in highly sensitive sequencing methods. We previously demonstrated that UDG pretreatment is efficacious for excluding nonspecific single nucleotide variants in amplicon-based MPS that occur if poor-quality DNA from FFPE samples was used. This may be a reason why the frequency of the PIK3CA mutation in our study was not higher than previous allele-specific mutation testing. Although the data on molecular profiling in this study was obtained from a validated comprehensive genomic assay, one of the limitations of this study is that our findings were not validated by other methods. Furthermore, because the sample size for each type of mutation was small, our results should be further validated in some independent cohorts in the future.

It is expected that PIK3CA mutations would imply poor clinical outcome, because the presence of oncogene activation leads to aggressive tumor behavior. However, this was not true. One possible hypothesis to explain the paradoxical result may be a negative feedback mechanism through which the PI3K/AKT pathway is inactivated in PIK3CA mutant tumors. In wild-type PIK3CA tumors, on the contrary, the PI3K/AKT pathway may be activated by several factors, such as EGFR and HER2, independent of PIK3CA mutation. Indeed, the relationship between PIK3CA mutation and p-AKT expression has been different among tumor types [19, 34]. Otherwise, wild-type PIK3CA tumors may require alternative molecular alterations to the PI3K/AKT pathway to acquire more aggressive phenotypes. However, all of them have not been proven yet, and our AQUA system did not include p-AKT expression. Therefore, such compensatory mechanisms need to be further elucidated in the future.

To further characterize the PIK3CA mutation and wild-type, we also investigated the clinicopathological characteristics of esophageal cancer patients with respect to PIK3CA status. PIK3CA mutations were significantly associated with the APC mutation. The type of APC gene alteration detected in this study was different from that reported to occur frequently in upper gastrointestinal cancers, such as at codons 1450, 1462–1465, and 1554–1556 [39]. The most frequently noted mutations in our cohort were nonsense mutations (11/17; 64.7%), which resulted in truncated APC proteins. APC frameshift deletions in the codon 1556 hot spot, 1301, and 1384 detected in this study also lead to loss of APC function. The coexistence of APC alterations with PIK3CA mutation may be partially explained by a previous study using a mouse model with PIK3CA mutation, which demonstrated that the PIK3CA mutation alone was insufficient to initiate intestinal tumorigenesis in intestinal cancers. Thus, loss of APC activity may synergistically act with active PI3K, resulting in tumorigenesis [40, 41].

As compared to PIK3CA alterations, the mutations involved in the RAS–RAF pathway were rare. Previous analysis by a mass-spectrometry based assays revealed that only one of 80 patients harbored an oncogenic BRAFV600E mutation [17]. In our series, no BRAFV600E mutation was detected. Instead, there were nine cases with BRAF mutations at codons 598 (*n* = 3), 469 (*n* = 2), and 444 (*n* = 2). We also found statistically significant coexistence of BRAF mutations and PIK3CA mutations. However, the biological relevance of the coexistence of these mutations remains unclear.

Furthermore, Zhang et al. successfully established and characterized patient-derived esophageal squamous cell carcinoma xenograft (PDECX) mouse models, and found that PDECX models with PIK3CA mutation had no significant response to cytotoxic agents. This result suggests that PIK3CA mutation is also involved in sensitivity to chemotherapy, and may provide an information for the treatment of ESCC patients in the future [42].

Recent treatment strategies for advanced esophageal cancer have shifted to neoadjuvant treatment, such as chemotherapy and chemoradiotherapy [3, 4]. The limitation of this study is that some of the surgically resected specimens may have been modified by preoperative therapy. Therefore, we also separately analyzed the survival for a cohort with preoperative treatment (*n* = 65) from that without preoperative therapy (*n* = 61). However, PIK3CA mutational status was not significantly associated with survival, probably because of the small sample size for PIK3CA mutations in each cohort. Furthermore, protein expression measured by AQUA may be modified by preoperative treatment. That could be a main reason why any gene expression was not significantly associated with prognosis in our study. Indeed, for instance, the prognostic effect of EGFR protein expression was proved by immunohistochemistry using surgically resected tumor tissue in patients with ESCC who underwent surgery alone or surgery and postoperative radiotherapy [43].

## Conclusions

Our study provides comprehensive genomic profiling of resected esophageal cancer by NGS using surgically resected FFPE tissue and identified PIK3CA mutations as a favorable prognosis biomarker. Our study supports previous findings obtained by allele-specific mutation testing. In the future, PIK3CA mutations may be potential targets in therapeutic development of esophageal cancer.

## Additional file

Additional file 1: All raw data on somatic mutational analysis by TSACP. (XLSX 38 kb)

## Abbreviations

AQUA: Automated quantitative fluorescent immunohistochemistry
ESCC: Esophageal squamous cell carcinoma
FFPE: Formalin-fixed paraffin-embedded
HR: Hazard ratio
MPS: Massively parallel sequencing
NGS: Next-generation sequencing
OS: Overall survival
qPCR: Quantitative PCR
TSACP: TruSeq Amplicon Cancer Panel
UDG: Uracil DNA glycosylase
